# A Case of Salivary Calculus

**Published:** 1855-05

**Authors:** H. K. Pusey

**Affiliations:** Garnettsville, Ky.


### ART. II.—A CASE OF SALIVARY CALCULUS.

BY H. K. PUSEY, M. D., OF GARNETTSVILLE, KY.

AN old black woman applied to me a few weeks since on account of a tumor on her jaw. The tumor was on the left side of the lower maxillæ, an inch below and half an inch in advance of the angle, and on examination was hard and did not appear to contain any fluid. The history given of the tumor by the patient was, that for the last thirty years she had, at times, been subject to swelling, pain, and tenderness about this part of her face, and that six weeks before she came to me the tumor began to form. It was then the size of a pullet's egg, tender to the touch, but without sensible fluctuation.

I directed the patient to apply a poultice and in a few days return and have the tumor punctured. The next day a spontaneous discharge of the contents took place, consisting of a small quantity of purulent matter and a smooth concretion an inch in length and three-eighths of an inch in diameter, of a pale-yellowish color, and weighing one hundred and forty grains. The body was fragile, pulverulent, and readily broke up into several pieces. It seems to be a *salivary calculus* formed in the duct of the parotid gland, which had been gradually increasing in size, and kept up irritation in the part for thirty years.

The growth of such bodies must necessarily be slow since the quantity of solid matter in the saliva is usually less than six parts in a thousand, of which only two-nineteenths are alkaline and earthy salts. The solid constituents of the saliva according to the analysis of FERICHS are ptyalin, mucus and epithelium, fatty matter, sulpho-cyanide of potassium, alkaline and earthy chlorides and phosophates, and oxide of iron. The composition of salivary calculi cannot vary materially from that of the saliva, and is indeed nearly identical with that of the "tartar" which collects on the teeth.

Concretions are not of so frequent occurrence in the salivary as in the lachrymal glands, but are nevertheless by no means uncommon. The most usual situation of these bodies is beneath the tongue, in the ducts of the sublingual glands, or in the duct of Wharton leading from the submaxillary gland. and the least common is the duct of Steno, of which only a few cases are on record. The case of which I have given the history above is to be added to the number. CRAIGIE mentions that these concretions are occasionally met with in the lower animals, and refers to instances in the mule and the ass, where calculi weighing six and thirteen drachms were found in the Stenonian duct.

It is only in rare instances that salivary concretions give rise to inflammation and ulceration, by which they are extricated, as happened in the case under my care. A case is recorded by SABATTIER in which the presence of a calculus in the duct of Wharton caused painful swelling of the submaxillary gland, thus giving rise to more suffering and inconvenience than the immediate swelling caused by the tumor. BOYER relates a case in which the submaxillary gland was inflamed and swelled, for which he performed an operation, extracting an oblong stony concretion, one extremity of which projected a little beyond the orifice of the duct of Wharton. An operation is generally necessary when these calculi become a source of annoyance.

ROKITANSKY, one of the latest writers on this subject and one of the most eminent pathological anatomists of the day, describes salivary calculi as "white, friable, and either round, oblong, cylindrical, or obovoid; in size varying from that of a millet-seed or pea, to even that of a hazelnut. They are either solitary, or, if small, frequently very numerous (twenty and more); and they are composed of phosphate and carbonate of lime, held together by animal matter."

It is a curious fact that while healthy saliva contains so little phosphate of lime salivary calculi should occasionally consist of ninety-four per cent. of that salt. Dr. WALSHE conjectures that "the excess of phosphate is generated through the influence of irritation of the mucous membrane;" but I see no reason for resorting to such a supposition. In the first place, it is not easy to understand how the "mucous membrane" can "generate" phosphate of lime. Salts are not generated by the animal tissues, but merely separated by them from the circulating fluid. Earthy and alkaline salts are constant ingredients of the saliva, and it is natural that they should constitute the bulk of the salivary concretions being much less soluble than the other ingredients. There is no disease of the mucous membrane of the bladder to generate the calcareous matter of urinary calculi, but, a nucleus presenting, the phosphates and other constituents of the urine readily precipitated, collect upon it, and gradually form the concretion.

*December*, 1854.

